# Serial Interval and Transmission Dynamics during SARS-CoV-2 Delta Variant Predominance, South Korea

**DOI:** 10.3201/eid2802.211774

**Published:** 2022-02

**Authors:** Sukhyun Ryu, Dasom Kim, Jun-Sik Lim, Sheikh Taslim Ali, Benjamin J. Cowling

**Affiliations:** Konyang University College of Medicine, Daejeon, South Korea (S. Ryu, D. Kim, J.-S. Lim);; Seoul National University, Seoul, South Korea (J.-S. Lim); The University of Hong Kong, Hong Kong, China (S.T. Ali, B.J. Cowling);; The Laboratory of Data Discovery for Health, Hong Kong Science and Technology Park, Hong Kong (S.T. Ali, B.J. Cowling)

**Keywords:** COVID-19, Delta variant, transmissibility, superspreading, South Korea, coronavirus disease, SARS-CoV-2, severe acute respiratory syndrome coronavirus 2, viruses, respiratory infections, zoonoses

## Abstract

We estimated mean serial interval and superspreading potential for the Delta variant of severe acute respiratory syndrome coronavirus 2 in South Korea. Intervals were similar for the first (3.7 days) and second (3.5 days) study periods. Risk for superspreading events was also similar; 23% and 25% of cases, respectively, seeded 80% of transmissions.

As of August 2021, South Korea is in the middle of a fourth community epidemic of severe acute respiratory syndrome coronavirus 2 (SARS-CoV-2) transmission, which is now predominated by the B.1.617.2 lineage (Delta variant) (*1*,*2*). The epidemic size largely depends on such epidemiologic characteristics as serial interval distribution and transmissibility (*3*,*4*). For the Delta variant of SARS-CoV-2, however, empirical evidence produced using country-level data are limited. We estimated serial interval distribution, reproductive numbers, and superspreading potential of SARS-CoV-2 during the Delta variant predominance in South Korea.

## The Study

**Figure 1:**
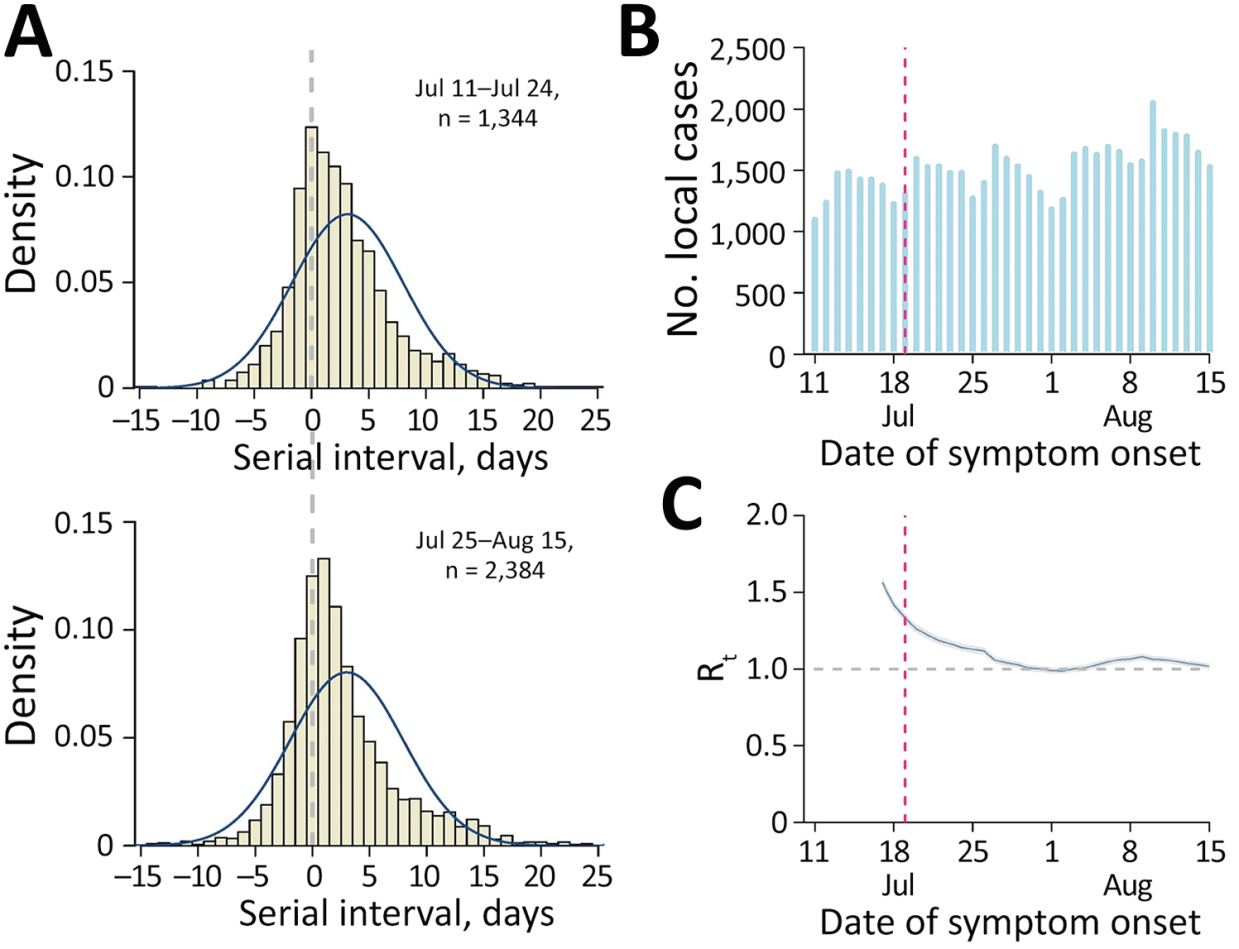
Estimated serial interval distribution, incidence of coronavirus disease, and transmissibility during predominance of the Delta variant of severe acute respiratory syndrome coronavirus 2 in South Korea. A) Estimated serial interval distribution for 3,728 infector-infectee pairs. Solid blue line indicates fitted normal distribution; vertical bars indicate the distribution of empirical serial intervals. B) Reported number of confirmed coronavirus disease cases by date of symptom onset. Red vertical dashed line indicates the date of implementation of an enhanced social distancing, including limiting gathering sizes to 4 persons nationwide on July 19, 2021. C) Estimated daily R_t_ of severe acute respiratory syndrome coronavirus 2 (blue line) with 95% credible intervals (gray shade). Gray horizontal dashed line indicates the critical threshold of R_t_ = 1. Red vertical dashed line indicates the date of implementation of an enhanced social distancing. R_t_, effective reproductive number.

We obtained line-list data on coronavirus disease (COVID-19) cases reported by South Korea local public health authorities during July 11, 2021–September 1, 2021. Because the detection rate of the Delta variant accounted for >50% of local cases after July 25, 2021, and to avoid right-censoring bias, we divided the study duration into 2 periods (period 1, July 11, 2021–July 24, 2021; period 2, July 25, 2021–August 15, 2021). Overall, 82,671 local cases were obtained during the whole study period; 19,635 cases were identified in period 1, and 34,569 cases were identified in period 2. The data included information on contact tracing with other reported cases of COVID-19 (i.e., the case number of infector or infectee) and dates of symptom onset. The serial interval represents the time between symptom onset for both the infector and the infectee in a transmission chain (*3*). On the basis of line-list information, we reconstructed the transmission pairs by identifying the infector and infectee. We identified 3,728 transmission pairs (1,344 pairs in period 1 and 2,384 pairs in period 2) having the date of symptom onset for both infector and infectee. The overall mean of the serial interval estimate was 3.6 days (95% credible interval [CrI] 3.5–3.6 days) and the SD of the serial interval estimate was 4.9 days (95% CrI 4.9–5.0 days). The mean serial interval estimate were 3.7 (95% CrI 3.5–3.8) days with an SD of 4.8 (95% CrI 4.8–4.9) days during period 1, and 3.5 (95% CrI 3.4–3.6) days with an SD of 5.0 (95% CrI 4.9–5.0) days during period 2 (Figure 1, panel A). We used Welch’s 2-sample t-test to compare the mean serial intervals for period 1 and period 2 and found no significant difference (p value = 0.40).

To identify the potential changes in SARS-CoV-2 transmissibility, we estimated the time-varying effective reproductive number (R_t_), which defines the mean number of secondary infectious cases generated from a typical primary infectious case at time *t*. The epidemic becomes under control if R_t_ falls below 1 sustainably. We estimated R_t_ by using the EpiEstim package in R (*5*). In South Korea, nonpharmaceutical interventions including a nationwide mask mandate have been implemented since 2020. Because a large number of COVID-19 cases were identified by mid-July 2021, a 4-person limit for gatherings was implemented beginning July 19, 2021, nationwide (*6*) (Figure 1, panels B and C). However, we identified that the estimated R_t_ was sustained at >1 during the study period (Figure 1, panel C).

**Figure 2:**
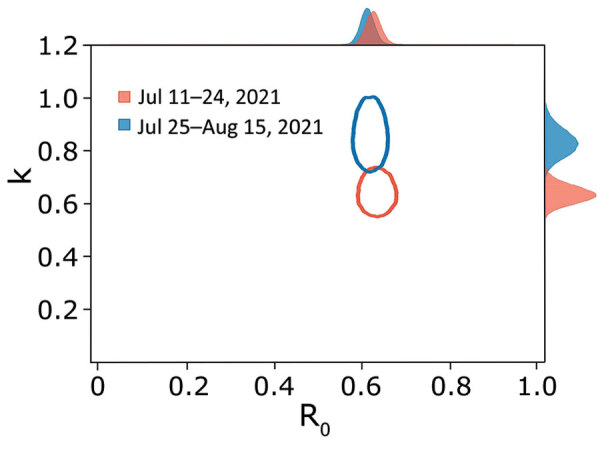
Risk for superspreading events for coronavirus disease during the Delta variant of severe acute respiratory syndrome coronavirus 2 predominance in South Korea. Joint estimates of *k* and R_0_ of coronavirus disease were calculated by using 5,778 pairs (2,169 for period 1 and 3,609 for period 2). The red and blue ovals indicate the bivariate 95% credible region of the estimated *k* and R_0_ for period 1 and period 2. The posterior marginal distributions were plotted in red and blue shaded regions. Period 1, July 11, 2021–July 24, 2021; period 2, July 25, 2021–August 15, 2021. *k*, overdispersion parameter; R_0_, basic reproduction number.

To analyze superspreading potential, we identified 5,778 transmission pairs that included the COVID-19 cases for which no date of symptom onset was provided for either infector or infectee (2,169 pairs for period 1 and 3,609 pairs for period 2). We calculated the number of secondary cases for each person from the transmission pairs and fitted the data into a negative binomial distribution (*7*) (Appendix). The 2 parameters of the distribution represent the reproduction number (R_0_) and overdispersion parameter (*k*). The estimated *k* for period 1 was 0.64 (95% CrI 0.57–0.72) and for period 2 was 0.85 (95% CrI 0.75–0.98), which corresponded to an expected percentage of cases responsible for 80% of secondary cases of 23% (95% CrI 22%–24%) for period 1 and 25% (95% CrI 24%–26%) for period 2 (Figure 2).

## Conclusions

We estimated the serial interval distributions of SARS-CoV-2 for early and later periods of the Delta variant predominance in South Korea and identified that mean serial intervals were similar across 2 different periods. This similarity is consistent with a recent study suggesting no substantial differences in the serial intervals between patients infected with the Delta variant and wild-type virus (*8*). In contrast, however, our findings suggested that the mean serial interval was 1 day longer than the estimates reported in a study describing the faster spread of the Delta variant in China (mean serial interval of 2.3 days) compared with the wild type (*9*). Changes to public health measures, such as active contact tracing and rapid isolation of COVID-19 patients, would have shortened the serial interval and reduced transmissibility and superspreading potential (*3*,*4*). Since June 10, 2020, however, the South Korean public health authority has consistently implemented strategies for active case finding and immediately isolating laboratory-confirmed COVID-19 patients and exposed persons by using digital QR codes (*10*). Therefore, the effect of enhanced case isolation against the serial interval of SARS-CoV-2 is likely limited in our study. Furthermore, restricting large gatherings had likely reduced the superspreading potential. However, because the R_t_ was >1 during most of the study period, the nonpharmaceutical interventions implemented were likely insufficient to control the transmission of SARS-CoV-2 in South Korea.

Our study’s first limitation is that we did not consider the effect of COVID-19 vaccinations in our analysis. About 14% of transmission pairs used in this study were linked with older adults (>60 years of age), who might have received COVID-19 vaccinations. However, the vaccination program was not implemented in members of the public <55 years of age in early August 2021. Second, we did not consider changes in nonpharmaceutical interventions on the local level and possible increased travel during the study period, because it included summer holidays. Enhanced social distancing, however, including limiting gatherings to 4 persons, was in place nationwide during the study period. Third, we retrieved online case reports, which could contain some inaccuracies. However, the daily number of laboratory-confirmed local cases was similar between the collected line list and official daily reports (Appendix). Last, because individual genotype information was not included in the line-list data, the proportion of the Delta variant was evaluated from alternative data retrieved from the Korea Disease Control and Prevention Agency.

A previous study from South Korea, which examined the early transmissibility of SARS-CoV-2 in February–March 2020, estimated the mean R_0_ as 1.5 for the wild type (*11*), and the early epidemic of COVID-19 was successfully controlled with nonlockdown social distancing (*12*). Our findings suggest that the introduction of the Delta variant is likely to have increased the difficulty of controlling SARS-CoV-2 transmission in South Korea. The large number of COVID-19 cases in South Korea during the study period could be explained by the increased secondary attack rate generated by cases of the Delta variant (*13*,*14*), which is in line with a previous study (*8*). Encouraging COVID-19 vaccination and further strengthening nonpharmaceutical interventions are warranted to mitigate spread of the Delta variant.

This article was preprinted at https://www.medrxiv.org/content/10.1101/2021.08.18.21262166v1.

Appendix Additional information about serial interval and transmission dynamics during SARS-CoV-2 Delta variant predominance, South Korea
