# Supplementary material for: Serial Interval and Transmission Dynamics during SARS-CoV-2 Delta Variant Predominance, South Korea
Source: Emerg Infect Dis. 2022 Feb;28(2):407–10. doi: 10.3201/eid2802.211774 (PMC8798673; doi:10.3201/eid2802.211774)
Supplement: Appendix — Additional information about serial interval and transmission dynamics during SARS-CoV-2 Delta variant predominance, South Korea [file 21-1774-Techapp-s1.pdf]

Article DOI: https://doi.org/10.3201/eid2802.211774

Serial Interval and Transmission Dynamics during SARS-CoV-2 Delta Variant Predominance, South Korea

Appendix

Data

We acquired data on coronavirus disease (COVID-19) cases, confirmed by real-time reverse transcription PCR (rRT-PCR) and reported by South Korea local public health authorities. The COVID-19 cases included occurred during July 11, 2021–September 1, 2021, when the Delta variant accounted for >40% of local cases reported from the Korea Disease Control and Prevention Agency (*1*,*2*). The data included contact tracing with other reported cases of COVID-19 and demographic characteristics of the patients, including age, sex, and date of symptom onset (https://github.com/gentryu/COVID-19delta).

Estimating Overdispersion Parameter (K)

Statistical methods used in this study followed our previous COVID-19 study for superspreading events (SSE) (*3*). The probability function of negative binomial distribution that an index case (i) generate the number of secondary case (y_i) is given by

$$Pr(Y = y_i) = \frac{\Gamma(k + y_i)}{y_i!\,\Gamma(k)}\left(\frac{k}{k + R}\right)^{k}\left(\frac{R}{k + R}\right)^{y_i},$$

where k and R are the estimated dispersion parameter and reproduction number, respectively, of index case i.

To understand the individual variation of infectiousness of COVID-19 during study period, observed offspring distributions were fitted to the negative binomial distribution. Given the estimated reproduction number and dispersion parameter, the proportion of infected persons responsible for 80% of secondary cases, $P_{80\%}$, was calculated by using equations from the previous studies (*3–6*). The proportion $P_{80\%}$ is given by

$$1 - P_{80\%} = \int_0^X NB\left(\lfloor x \rfloor; k, \frac{k}{R + k}\right) dx,$$

where X satisfies

$$1 - 0.80 = \frac{1}{R}\int_0^X \lfloor x \rfloor \, NB\left(\lfloor x \rfloor; k, \frac{k}{R + k}\right) dx.$$

Furthermore, with the threshold for SSE as 6 secondary cases defined in the previous study (*4*), the proportion of SSE was estimated for each period with the equations below.

$$\int_6^\infty NB\left(\lfloor x \rfloor; k, \frac{k}{R + k}\right) dx = 1 - \int_0^5 NB\left(\lfloor x \rfloor; k, \frac{k}{R + k}\right) dx.$$

Finally, using the branching process, the expected probability that 1 index case of SARS-CoV-2 infection results in a cluster of size s were estimated with the equations from previous studies (*3*,*5*,*6*) given by

$$r_s = \frac{\Gamma(ks + s - 1)}{\Gamma(ks)\Gamma(s + 1)}\left(\frac{R}{k}\right)^{s-1}\left(\frac{k}{k + R}\right)^{ks + s - 1},$$

where the probability of cluster of size s or greater could be estimated as follows:

$$p_s = 1 - \sum_{l=1}^{s-1} r_l.$$

With the condition that N seed cases were introduced into the totally susceptible populations, the estimated probability that ≥1 cluster of size s or greater occurs is

$$P_{N,s} = 1 - (1 - p_s)^N.$$

Note that $P_{N,s}$ is equal to p_s when exactly 1 index case was introduced ($N = 1$).

We used a Bayesian Markov Chain Monte Carlo simulation using *rstan* package. Four chains of 40,000 iterations were obtained with 5,000 burn-in. The prior distributions of dispersion parameter and reproduction number were uniform with lower and upper bounds set at 0 and 100. Convergence was checked visually by using a trace plot and the Gelman–Rubin–Brooks diagnostic (*7*). The posterior distribution of the estimates was demonstrated with the median and 95% credible intervals (CrI).

Supplementary Analysis for the Risk for Superspreading Events

We estimated the expected proportion of cases responsible for 80% of the total secondary cases to identify the risk of SSE. Furthermore, we estimated the probability of SSEs using estimated R_0 and k and the probability that 1 index case results in a cluster of ≥10 cases.

Based on the source of infection, we estimate the offspring distribution using infectee-infector pairs. We fitted observed distribution of the secondary case into a negative binomial offspring distribution (Appendix Figure 1). The expected proportion of cases responsible for 80% of secondary cases was 22.99% (95% CrI 21.97%–24.03%) for period 1 and 24.93% (95% CrI 23.90%–25.97%) for period 2. The probability of SSE and the probability that 1 index case results in a cluster of ≥10 cases were 0.33% (95% CrI 0.25%–0.45%) and 4.71% (95% CrI 3.92%–5.60%) for period 1, and 0.17% (95% CrI 0.12%–0.24%) and 4.254% (95% CrI 3.51%–5.11%) for period 2.

References

1. Cha S. South Korea reports record daily infections as Delta variant drives surge. 2021 Jul 21 [cited 2021 Aug 6]. https://www.reuters.com/world/asia-pacific/south-korea-reports-new-daily-record-1784-covid-19-cases-kdca-2021-07-21

2. Korea Disease Control and Prevention Agency. COVID-19 update as of 3 August 2021. 2021 [cited 2021 Aug 6]. https://www.korea.kr/news/policyBriefingView.do?newsId=156464695#sitemap-layer

3. Lim JS, Noh E, Shim E, Ryu S. Temporal changes in the risk of superspreading events of coronavirus disease 2019. Open Forum Infect Dis. 2021;8:ofab350.

4. Adam DC, Wu P, Wong JY, Lau EHY, Tsang TK, Cauchemez S, et al. Clustering and superspreading potential of SARS-CoV-2 infections in Hong Kong. Nat Med. 2020;26:1714–9. PubMed https://doi.org/10.1038/s41591-020-1092-0

5. Endo A, Abbott S, Kucharski AJ, Funk S; Centre for the Mathematical Modelling of Infectious Diseases COVID-19 Working Group. Estimating the overdispersion in COVID-19 transmission using outbreak sizes outside China. Wellcome Open Res. 2020;5:67. PubMed https://doi.org/10.12688/wellcomeopenres.15842.3

6. Kucharski AJ, Althaus CL. The role of superspreading in Middle East respiratory syndrome coronavirus (MERS-CoV) transmission. Euro Surveill. 2015;20:14–8. PubMed https://doi.org/10.2807/1560-7917.ES2015.20.25.21167

7. Gelman A, Carlin JB, Stern HS, Dunson DB, Vehtari A, Rubin DB. Bayesian data analysis. Boca Raton (FL): Chapman and Hall/CRC Press; 2013.

Appendix Table 1. Age-specific distribution of SARS-CoV-2 infector-infectee pairs having symptom onset for both infector and infectee, South Korea*

Age group of infector, y	Age group of infectee, y								
	0–9	10–19	20–29	30–39	40–49	50–59	≥60	NA	Total
0–9	16	16	26	32	20	34	8	1	153
10–19	18	51	82	57	78	45	42	0	373
20–29	48	76	160	119	130	126	52	3	714
30–39	41	57	134	133	103	87	34	2	591
40–49	41	67	112	84	103	115	49	1	572
50–59	34	47	125	96	103	113	60	2	580
≥60	21	31	61	43	43	44	38	1	282
NA	0	3	2	0	1	5	2	450	463
Total	219	348	702	564	581	569	285	460	3,728

*NA, not available; SARS-CoV-2, severe acute respiratory syndrome coronavirus 2.

Appendix Table 2. Age-specific distribution of SARS-CoV-2 infector-infectee pairs for whom no date of symptom onset was reported for either infector or infectee, South Korea*

Age group of infector, y	Age group of infectee, y								
	0–9	10–19	20–29	30–39	40–49	50–59	≥60	NA	Total
0–9	36	31	46	58	41	66	19	4	301
10–19	37	62	107	80	109	79	60	0	534
20–29	86	107	234	170	196	179	82	4	1,058
30–39	73	87	195	188	144	136	73	3	899
40–49	64	103	159	127	161	187	75	3	879
50–59	71	84	188	151	139	192	104	3	932
≥60	29	53	117	71	67	79	68	2	486
NA	0	4	7	0	1	6	4	667	689
Total	396	531	1,053	845	858	924	485	686	5,778

*NA, not available; SARS-CoV-2, severe acute respiratory syndrome coronavirus 2.

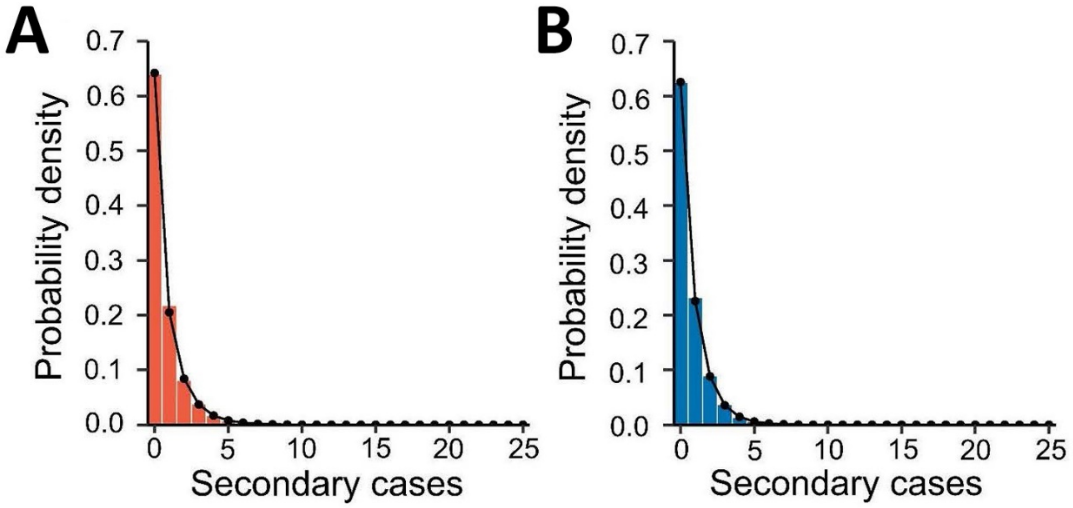

Appendix Figure 1. Distributions of the number of secondary cases of coronavirus disease in A) period 1 (July 11, 2021–July 24, 2021) and B) period 2 (July 25, 2021–August 15, 2021) and corresponding fitted negative binomial distributions, South Korea.

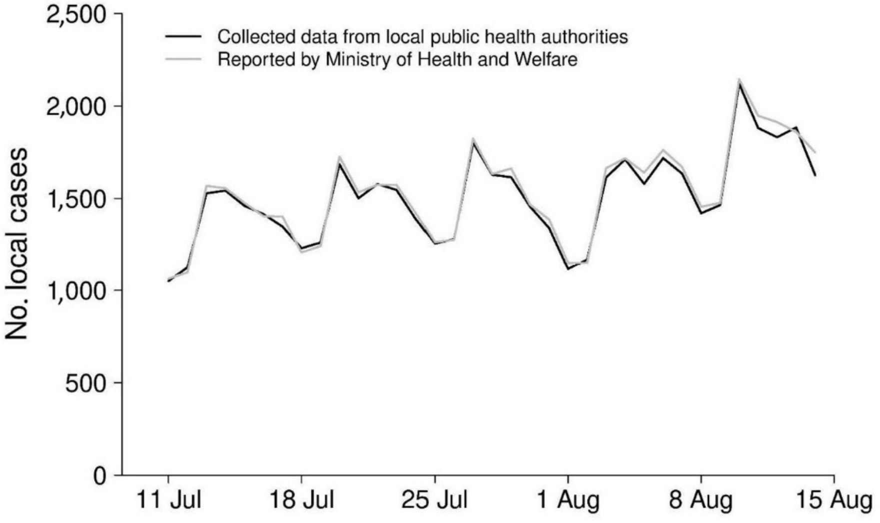

Appendix Figure 2. Daily number of coronavirus disease cases from the collected data and reported number by the Korean Ministry of Health and Welfare, South Korea. The black line indicates data collected from the local public health authority. The gray line indicates the daily number of coronavirus disease cases reported by the South Korean central government.
